# The test-retest reliability, concurrent validity, and minimal detectable change of the *L* test in patients with total hip arthroplasty

**DOI:** 10.1186/s43161-021-00038-8

**Published:** 2021-08-25

**Authors:** Fatih Özden, Gökhan Coşkun, Serkan Bakırhan

**Affiliations:** 1 Köyceğiz Vocational School of Health Services, Elderly Care Department, Muğla Sıtkı Koçman University, Köyceğiz, Muğla Turkey; 2 Faculty of Health Sciences, Physiotherapy and Rehabilitation Department, Ege University, Bornova, İzmir, Turkey; 3 Home Health Care Services, Adana City Training and Research Hospital, Yuregir, Adana Turkey

**Keywords:** *L* test, Reliability, Total hip arthroplasty, Validity

## Abstract

**Background:**

The *L* test is a modified version of the timed up and go test (TUG) with an L-shaped walking path. The *L* test is more extensive than other performance tests, especially in turn direction and specific tasks. The study aimed to evaluate the test-retest reliability, concurrent validity, and minimal detectable change of the *L* test in patients with total hip arthroplasty (THA). A cross-sectional study was conducted with 33 unilateral and primary THA patients. The *L* test was performed twice with an interval of an hour on the same day for the test-retest reliability. Timed up and go test (TUG), five times sit to stand test (FTST), and Harris hip score (HHS) were carried out for the analysis of the concurrent validity of the *L* test.

**Results:**

The mean age of the participants was 74.6 ± 10.3 years. The ICC score of the *L* test was 0.992. Test-retest reliability was excellent. SEM_95_ and MDC_95_ values were 3.39 and 9.39, respectively. Both TUG and HHS were strongly correlated with the *L* test (*r*_1_ = 0.889, *r*_2_ = −0.568, *p* < 0.001). However, there was no significant correlation between FTST and *L* test (*r* = 0.024, *p* > 0.05).

**Conclusions:**

The *L* test is valid and reliable performance measurement in patients with primary unilateral THA. MDC_95_ of the *L* test is an essential reference for clinicians in the rehabilitation follow-up process of THA patients.

## Background

Total hip arthroplasty (THA) is a common surgical procedure used to prevent pain, joint instability, physical activity limitation, and gait disturbances caused by hip osteoarthritis [1, 2]. Walking is closely related to the whole functional level as it has very high importance for daily life and is an essential determinant of functional recovery in patients with THA [3]. Assessment of walking in patients with THA is essential in indicating the improvement and increase in functional level in the postoperative period of the surgery [4]. Although improvement is achieved in the walking parameters following the THA surgery, walking disorders continue compared to healthy people [3].

Reliable and valid standardized performance tests provide the evaluation and monitoring of the gait [5]. In particular, performance-based testing can provide significant benefits in terms of being easy to use, practical, simple, and cost-free for research, clinical examination, and gait analysis [6]. Different tools such as the 10-m walk test, 50-foot walk test, timed up and go test (TUG), and 2-min walk tests are used to assess walking speed, turns, or walking endurance in patients with THA [4, 7, 8]. For instance, the 10-m walk test is widely used, valid, and reliable in orthopedic rehabilitation. However, these tests do not evaluate essential parameters of daily activities, such as turning left and right or transfer activities. The 2-min or 6-min walking tests measure walking endurance; however, these physical performance tests involve too much duration and available field to perform [9].

The TUG is a highly valid, reliable, and practical tool to evaluate physical activity. However, this test allows the patients to return from the preferred direction and has a shorter walking distance (6 m) [10]. The *L* test is a more complex and extended version of the TUG test. *L* test renewed the gait interval from 6 to 20 m and allowed patients to turn clockwise and counterclockwise. Therefore, potential floor or ceiling effects could be prevented, and also measurement’s limited sensitivity could be overcome [11]. The TUG does not fully demonstrate THA components’ physical capacities and it remains limited in determining their capacity. Another feature of the *L* test is that it requires a better ability in both left and right turns and the transition to a sitting position. The *L* test also allows patients to be tested in a hospital or home setting with rooms and corridors [12]. In patients with THA, it is necessary to identify practical and more functional assessment methods that preferably externalize actual conditions to evaluate performance, especially walking. It may be advantageous to evaluate elderly patients who receive residential care in a home environment. A recent study showed no difference between home and clinical settings for the performance-based test measurement [13]. Besides, elderly patients with THA may not show in average performance due to fatigue and anxiety in the hospital. In unusual conditions such as COVID-19, *L* test could provide an excellent convenience in evaluating patients during remote health care and home care services [14].

To our knowledge, no other study investigated the psychometric properties of the *L* test in patients with THA. The study was aimed to reveal the test-retest reliability, concurrent validity, and minimal detectable change of the *L* test in patients with THA.

## Methods

### Setting and participants

A cross-sectional study was conducted in Adana City Training and Research Hospital, at the Department of Home Health Care. All of the measurements were carried out in the patient’s home. Our study was designed prospectively with the “test-retest method.” A total of 33 unilateral primary THA patients were enrolled in the study. All patients were at their 6-month follow-up post THA surgery and were clinically stable. The patients were eligible if they had THA surgery 6 months ago and ≥ 18 years. The exclusion criteria of the study were as follows: (1) had bilateral THA, (2) had revision THA, (3) other lower extremity surgeries, (4) cognitive impairment, (5) neurological or orthopedic conditions that may affect walking, (6) patients with visual impairment, (7) unconsented participants. The research has conformed to the principles of the Helsinki Declaration. Patients’ consent was given. The ethics committee of Ege University (No:20-7T/77) approved the research.

The required sample size was calculated with the G*power 3.1 (*f* = 0.4, alpha level = 0.05, and power = 0.80) [15]. According to our calculation, it was sufficient to perform statistical analyses with at least 34 patients. Accordingly, we conducted our study with 33 THA patients.

### Experimental procedure

All of the measurements were taken by the same physiotherapist. In this way, it was aimed to avoid measurement errors due to variations between evaluators [16]. Evaluations were conducted only with participants who had the appropriate room and corridor length/width in their home setting. We conducted our study in the home environment to prevent the potential abnormal walking speed of the patients caused by fatigue and anxiety during the hospital admission and transfer period. The home environment can better represent functional levels in everyday activities, as these elderly THA patients are entirely home residents. Besides, the *L* test developers stated that the test is spontaneously developed while in the training session with their patients. They claimed that they were asked by their patients to “walk 3 m from the clinic’s room and go out by walking 7 m in the corridor, then come back from the same pathway.” We considered that this test could be applicable in the home setting with the same situation in the home and can be of great benefit, especially for older THA patients. Accordingly, the room and the corridor were standardized in all tests before the applications to create a standard setting.

In all of the participants’ home settings, there were no obstacles that could negatively affect the patients’ gait speed. Rooms and corridors without any materials that could also hinder gait speed, such as carpets, were preferred. The stopwatch was used to record the elapsed time in seconds during the test. The test was applied once in both test and retest, especially considering that the patients are elderly. It was aimed to prevent effects such as walking endurance, fatigue, and learning effects. All the participants were provided with practical information about the test. In this manner, the patients were given to learn the tests well before applying. Patients were asked to walk as quickly as possible but safely and comfortably during the test. The participants were given verbal instructions during the test; however, these instructions were not intended to encourage the patient. Patients were allowed to walk with assistive devices that they continuously use to observe their actual physical performance. During the 1 h between test and retest, patients were only allowed to drink water. Patients were seated between the test and re-test to prevent the fatigue effect [17].

### Outcome measures

The sociodemographic, physical, and clinical characteristics primarily related to the gait of patients were recorded. In addition, the pain status during rest and gait was questioned with a visual analog scale (VAS) [18]. Timed up and go test (TUG), five times sit to stand test (FTST), and Harris hip score (HHS) was carried out for the analysis of the concurrent validity of the *L* test [19]. The *L* test was performed twice with an interval of an hour on the same day for the test-retest reliability.

#### *L* test

The *L* test is an extended and comprehensive version of the TUG test regarding walking distance and mandatory turning directions. It provides a more comprehensive physical performance evaluation than TUG. In this test, individuals are asked to get up from the chair, walk up to the cone located 3 m away, and turns 90 degrees to the right. After walking ahead 7 m, the patient turns 180 degrees around a cone, returns from the same pathway, and sits on the chair. The time elapsed during the test was recorded [6, 12]. The elapsed time during the test was not reported to the patient.

#### Timed up and go test (TUG)

TUG is a highly preferred test in evaluating physical performance, especially in orthopedics and traumatology after surgery. During the test, essential movement components of the individuals are evaluated. The patient sits in a standard chair. With the command to start, the patient stands up, walks up to the line 3 m from the chair, turns back, and sits back on the chair. The test is performed at average walking speed. The time begins when the patient’s back is no longer in contact with the chair. The test is concluded when the patient’s back makes full contact with the chair. The time taken is the score of the test [20].

#### Five times sit to stand test (FTST)

FTST evaluates the individual’s sitting and standing activities by focusing more on lower extremity muscle strength. The participants sit with their hands on their shoulders and arms crossed. The patient’s five times sit-to-stand duration was recorded as the score of the test [21].

#### Harris hip score (HHS)

HHS is one of the most commonly used measurement tools in hip osteoarthritis, before and after hip arthroplasty, and in assessing patients’ functional status. In this scoring system, the clinician conducts a comprehensive evaluation, such as pain, deformity, range of motion, and daily activities. The patient is scored out of 100. A higher score indicates a better clinical condition. The Turkish version of THA has been validated and shown to be reliable [19].

#### Statistical analysis

Statistical analyses were calculated by “SPSS for Windows v25.0” (SPSS Inc., Chicago, IL, USA). Quantitative variables were presented as mean and standard deviation (SD). For qualitative variables, a percentage (%) was given. Kolmogorov–Smirnov tests were used to calculate the normal distribution of data. The confidence interval of 95% was accepted. ICC is graded as fair (0.60-0.80) and high (0.80-1.0), considering statistical recommendations [22].

The minimal detectable change (MDC) and standard error of measurement (SEM_95_) were calculated as follows [23]:

$$ {\mathrm{MDC}}_{95}=1.96\ast \mathrm{SEM}\ast \surd 2 $$

$$ \mathrm{SEM}95=\mathrm{SD}\ast \surd \left(1-\mathrm{ICC}\right) $$

Pearson correlation coefficient was used to assess the validity. The concurrent validity of the *L* test was evaluated with HHS, FTST, and TUG. The correlation coefficient was classified as follows: ≥ 0.5 (strong), 0.5-0.35 (moderate), ≤ 0.35 (poor) [24].

## Results

The mean age of the patients was 74.6 ± 10.4 years. Twenty-one women (63.6%) and 12 men (36.4%) were both tested and retested. The sociodemographic, physical, and clinical specifications of the participants are given in Table 1. The absolute values of the clinical assessments were presented in Table 2. The mean pain severity of the patients at rest and walking were 2.09 ± 1.95 and 3.27 ± 2.38, respectively. The mean time of the second test was 3.55 s better than the first assessment. Reliability and validity analysis of the *L* test are presented in Tables 3 and 4, respectively. The ICC score of the *L* test was 0.992. Test-retest reliability was excellent. SEM_95_ and MDC_95_ values were 3.39 and 9.39, respectively. Both TUG and HHS were strongly correlated with the *L* test (*r*_1_ = 0.889, *r*_2_ = −0.568, *p* < 0.001). However, there was no significant correlation between FTST and the *L* test (*r* = 0.024, *p* > 0.05).

**Table 1:** The sociodemographic, physical, and clinical characteristics of the patients

*n*: 33	Total
Age (years, mean ± SD)	74.6 ± 10.4
BMI (kg/m^2^, mean ± SD)	27.4 ± 3.3
VAS	
Rest	2.09 ± 1.95
Walking	3.27 ± 2.38
Gender (*n*, %)	
Women	21 (63.6)
Men	12 (36.4)
The operated side (*n*, %)	
Right	18 (54.5)
Left	15 (45.5)

*SD* standard deviation, *n* number of patients, *VAS* visual analog scale

**Table 2:** Absolute values (mean, standard deviation, min-max) of the evaluations

*n*: 33	Mean ± SD	Range
TUG	27.00 ± 10.47	(12-48)
FTST	25.09 ± 8.55	(12-40)
HHS	64.80 ± 13.77	(44.65-93)
*L* test		
Test	91.03 ± 38.70	(21-152)
Retest	87.48 ± 38.16	(18-147)

*SD* standard deviation, *n* number of patients, *TUG* timed up and go test, *FTST* five times sit to stand test, *HHS* Harris hip score

**Table 3:** Test-retest reliability of the *L* test

***n***: 33	Difference (mean ± SD)	ICC (95% CI)	SEM_**95**_	MDC_**95**_
***L*test**	3.54 ± 4.84	0.992 (0.98-0.99)	3.39	9.39

*n* number of patients, *ICC* intra-class correlation coefficient, *CI* confidence interval, *α* Cronbach’s alpha, *SEM* standard error of measurement; *MDC* minimal detectable change

**Table 4:** Correlation coefficient between *L* test with TUG, FTST, and HHS

***n***: 33	***L*** test (test)
**TUG**	0.889**
**FTST**	0.024
**HHS**	−0.568**

** *p* < 0.001, *n* number of patients, *TUG* timed up and go test, *FTST* five times sit to stand test, *HHS* Harris hip score

## Discussion

*L* test is a reliable and valid performance test for the evaluation of patients with THA. Besides, MDC values of the *L* test have been demonstrated in THA patients. This value provides essential data for clinicians studying orthopedic rehabilitation to monitor post-surgical patients’ clinical status changes. By revealing the *L* test’s psychometric values, a specific performance test has been introduced, in which THA patients can be evaluated in a standardized way. This test is an assessment tool that has been used in many case groups for many years, effectively representing the patient’s daily life activities with sitting, standing, walking, and changing direction [25]. It modifies the TUG test and consists of necessary mobility tests that measure walking speed in seconds [11, 26]. TUG does not fully reflect the patient’s functions during in-home or other daily life activities. Accordingly, the TUG test is insufficient in evaluating patients’ performance levels with THA, which includes more walking distance and balance parameters. Furthermore, the TUG test only includes turning back. The *L* test involves both turning back, right, and left. This specification provides the ability to reveal hidden symptoms that TUG cannot fully detect in patients with unilateral involvement [25]. Our study used the *L* test to evaluate rotational activities in more detail in patients with unilateral involvement THA.

ICC value was found to be 0.992 (95% CI = 0.98-0.99). Test-retest reliability was excellent. The ICC score of the *L* test’s test-retest reliability was found to be 0.97 in three separate studies conducted with older adults, Parkinson’s disease, and individuals with lower extremity amputation [6, 11, 26]. In the *L* test’s reliability study with stroke patients, ICC was calculated 0.99, similar to our study [25]. Our study shows similar results with the reliability of other studies. The *L* test was found reliable in patients with THA as in other case groups.

The *L* test was compared with HHS, a performance-based scoring system, using a Pearson correlation coefficient. The correlation coefficient was −0.568. The relationship between HHS and the *L* test was strong. Considering that HHS is a common scoring system that evaluates the functions of daily living activities such as sitting, sitting and walking, comparison with the *L* test gave accurate results in the group of patients with THA [19]. Also, the *L* test was strongly correlated with TUG (*r* = 0.889). This result was expected, considering that the *L* test was a modified version of the TUG. However, the *L* test was not related to FTST (*r* = 0.024). The FTST does not include a walking task and primarily represents lower extremity muscle strength [21]. Because the *L* test requires much more dynamic balance capability than FTST, we interpreted that the two tests might not yield similar results in terms of these features. Therefore, they might not be correlated.

In our study, SEM_95_ and MDC_95_ values were found 3.39 and 9.39, respectively. These values provide a significant contribution to the clinical practice and research. In the evaluation of the patient, SEM was used to identify the situations related to possible errors and variations obtained from the *L* test, and MDC was used to interpret the minimum level of clinical significance for the score achieved by the THA patient [23]. Clinicians should expect at least 9.39 s of development from the *L* test to observe the effectiveness of their therapy applications within the scope of orthopedic rehabilitation. In this way, a more precise and realistic clinical evaluation can be made with the *L* test.

Limitations of the study should be acknowledged. The MDC value found in our study may not be generalizable for the more active and relatively young THA patient group, given that our case sample is elderly individuals receiving home care services. Secondly, examining other kinematics such as acceleration and speed in laboratory conditions using the inertial sensor will provide a comprehensive analysis opportunity.

## Conclusion

*L* test is a valid and reliable performance test in patients with THA. The test-retest reliability and concurrent validity of the *L* test were excellent. *L* test is a practical, cost-free, and valuable tool for evaluating patients in their home or natural setting. Clinicians will be able to more effectively observe the clinical change in the postoperative rehabilitation phase of patients using the MDC value of the *L* test.

## Data Availability

Not applicable.

## Abbreviations

BMI: Body mass index
CI: Confidence interval
FTST: Five times sit to stand test
HHS: Harris hip score
ICC: Intra-class correlation coefficient
MDC: Minimal detectable change
THA: Total hip arthroplasty
TUG: The timed up and go test
SD: Standard deviation
SEM: Standard error of measurement
*n*: Number of patients
*r*: Pearson correlation coefficient
*α*: Cronbach’s alpha
